# The Livestock Frontier in the Paraguayan Chaco: A Local Agent-based Perspective

**DOI:** 10.1007/s00267-024-01957-7

**Published:** 2024-03-08

**Authors:** María José Milán, Elizabeth González, Feliu López-i-Gelats

**Affiliations:** 1 Department of Animal and Food Science, Universitat Autònoma de Barcelona, Bellaterra, 08193 Spain; 2 Agroecology and Food Systems Chair, Universitat de Vic-Universitat Central de Catalunya, Vic, 08500 Spain

**Keywords:** Q methodology, Agricultural frontier, Tropical deforestation, Cattle ranching, Discourse

## Abstract

Deforestation is one of the most relevant transformations characterizing global environmental change in the tropics at present. There is wide consensus in pointing the context-dependent nature of tropical deforestation. In this sense, a better characterization of the phenomenon considering the social context could provide a more accurate picture of tropical deforestation. With this aim, a Q-methodology discourse analysis was conducted to characterise the different discourses that coexist in the particular region of the Paraguayan Chaco concerning the development of cattle ranching and derived deforestation. Four different discourses were identified as making sense the wide range of interests and values coexisting and clashing in the Paraguayan Chaco, namely: the Environmentalist discourse, the Business discourse, the Resigned discourse, and the Possibilist discourse. The results point that the fundamental differences between the discourses are largely explained by the different positions on three specific domains: (i) the socio-economic benefits the expansion of cattle ranching brings about; (ii) the environmental impacts the expansion of cattle ranching and the derived deforestation brings on; and, finally (iii) the degree to which an active intervention from the side of policy making to regulate the expansion of cattle ranching and to minimize possible detrimental effects is seen as necessary. The position of the different discourses in relation to these domains could help policy makers to make measures and regulations more widely accepted and followed.

## Introduction

Deforestation is one of the most dramatic transformations characterizing global environmental change at present. Most of the deforestation is taking place in the tropics and at alarming rates, particularly in Southeast Asia and in South America (Rudel et al., 2009; van Vliet et al., 2012; Austin et al., 2017). Despite long evidence that tropical deforestation remains one of the most notable transformations undergoing on Earth, and that there is wide consensus in pointing its context-dependent nature (Rudel, 2007; Zak et al., 2008; Caldas et al., 2015; le Polain de Waroux et al., 2016; 2018; Meyfroidt et al., 2018; Piquer-Rodríguez et al., 2018), accurate characterization of the social context has been poorly considered in these studies. This is the case despite the fact that: (i) the debate about how to harmonize food production with the conservation of the environment (Perfecto and Vandermeer 2008) and the landscape, as well as the rights of indigenous and local communities (Zepharovich et al., 2020a) is booming; and (ii) the growing recognition that nature conservation requires social endeavour (Bennett et al., 2017; Zabala et al., 2018) and that it is fundamental a better comprehension of the values and interests people ascribed to the conservation of natural resources in different contexts (Bennett 2016; Bennett et al., 2017; Cáceres et al., 2020).

The expansion of land for agricultural use and the consequent deforestation, together with society’s increasing environmental concerns, are the cause of multiple conflicts (Cáceres et al., 2020; Zepharovich et al., 2020a, b; De Jong et al., 2021). These conflicts are often reflected in the media and in debates in a very simplistic and even dichotomous way: “conservationists or environmentalists” versus “productivists” (Miller et al., 2011; Hoelle, 2018; Huaranca et al., 2019). These dichotomous positions are sometimes self-serving and underlie divergent interests and values among the parties, usually in a context of power asymmetries (Robbins, 2006). Moreover, they can overshadow other insights that help to understand how society makes sense of this phenomenon (Hajer and Versteeg, 2005) and to anticipate in which way the policy may be received positively and in which way it is contrary to the objectives and beliefs of the farmers (Davies and Hodge, 2007).

Consideration of the prevailing perceptual frames in a given conflict, considering all its complexity, is crucial to examine the motivations, attitudes and consequences that underlie a given phenomenon (Hajer and Versteeg, 2005; Huaranca et al., 2019). Identifying and delving into different perceptions can reveal unexpected insights, marginalized points of view and potential points of consensus (Brannstrom, 2011; Zabala et al., 2018; Huaranca et al., 2019; Zepharovich et al., 2020b), which can be useful in resolving conflicts. This knowledge can also prove useful in identifying the underlying causes of lack of support for certain initiatives, as well as characterizing the attributes that a given intervention must meet to secure long-term support (Davies and Hodge, 2007; Zabala et al., 2017). One of the ways to study social perceptions is by capturing the coexisting discourses on a given topic (Webler et al., 2001). Hajer and Versteeg (2005) and Robbins (2006) agree on the importance of analysing discourses in environmental governance studies. For these authors, discourses are important precursors to creating coalitions among diverse actors with the aim of resolving complex environmental conflicts. Webler et al. (2001) and Robbins (2006) suggested that the resolution of political debates occurs through discursive coalitions that are they configure complex systems of power and knowledge that also form and reproduce identity, so the empirical study of discourses allows a better understanding of the relations between knowledge and power in relation to the formulation of environmental policies.

Our work is motivated by the hypothesis that a better characterization, in social terms, of tropical deforestation could provide a more accurate picture of the phenomenon. The premise behind this article is that there is more variety than uniformity among the landowners and other agents involved in cattle ranching, in frontier forest regions, such as the Paraguayan Chaco. In view of this, the purpose of this paper is to describe and attend to these differences in attitudes between stakeholders because they might point to ways to reduce rates of deforestation without angering the frontier forest farmers who do so much of the land clearing. To do so we employ the Q methodology discourse analysis to examine the coexisting attitudes towards land use changes. The application of this methodology to measure social perspectives on issues related to sustainability and environmental governance (Brannstrom 2011; Zabala et al. 2018; Walder and Kantelhardt 2018; Pinillos et al., 2021; Sneegas et al., 2021) has grown considerably in the last two decades. More specifically, in the Argentine Chaco three recent studies have been carried out to identify and examine social perceptions of local agents on deforestation (Huaranca et al., 2019; Zepharovich et al., 2020b), from the perspective of environmental justice (Zepharovich et al., 2020a) and in relation to ecosystem services (Córdoba and Zepharovich, 2022). In view of this, the objective of this paper is to explore the multiple discourses coexisting among local agents, directly or indirectly involved in cattle ranching, about the causes and implications of deforestation in the Paraguayan Chaco and the role that cattle ranching plays in the deforestation. In this work, we address the following questions: What do local agents think about the expansion of cattle ranching in the Paraguayan Chaco? What advantages and risks do local agents see in this expansion? What are their views on land use change and the resulting deforestation? and What are the underlying motivations of local agents to behave as they do?.

### Case Study: Cattle Ranching and Deforestation in the Paraguayan Chaco

Over the last two decades, the Gran Chaco, which is the largest biome in South America after the Amazonia, has experienced some of the highest rates of deforestation in the world, with a total of 14 million ha of forest (12% of the territory) being converted to agricultural land between 1985 and 2013 (Graesser et al., 2015; Baumann et al., 2016). The tropical dry forests of the Gran Chaco region, comprising parts of Eastern Bolivia, Northern Argentina, Southwestern Brazil and Western Paraguay, have become a hotspot of deforestation as a consequence of the expansion of soy cultivation and cattle ranching (Nepstad et al., 2006; Barona et al., 2010; Gasparri and le Polain de Waroux, 2015; Fehlenberg et al., 2017; Cáceres et al., 2020). More specifically, in the Paraguayan Chaco, an annual rate of deforestation of 1.0% was reported between 1987 and 2012, with a total loss of 44,000 km^2^ of forest (Baumann et al., 2017). Salinas et al. (2023) report a deforestation rate of 32.3% between 1999 and 2021. Fundamentally, land use is changed from forest to grassland for animal feeding (Caldas et al. 2015; Baumann et al., 2016; 2017). The rate of deforestation more than doubled between 2001 and 2012 compared to the one observed between 1987–2000 (Baumann et al., 2017), being at present one of the most active deforestation frontiers in the world. The main cause of deforestation in the Paraguayan Chaco is the expansion of cattle ranching; in this region, cropland expansion only played a minor role (Graesser et al., 2015; Baumann et al., 2017), like what was observed in Amazonia (Margulis, 2004).

As cattle ranching developed, the region has undergone notable transformations, such as increased use of highly productive exotic grasses like Gatton panic and Buffel grass and new cattle breeds, as well as changes in the land ownership structure (Baumann et al., 2016; 2017), resulting in a high environmental and socioeconomic impact. However, unlike other regions of the Gran Chaco, where studies have been conducted on the social and environmental impact of agricultural and livestock expansion (Cáceres et al., 2010; 2015; 2020; Marinaro and Grau, 2015; Huaranca et al., 2019; Zepharovich et al., 2020a; Córdoba and Zepharovich, 2022), studies of this nature are lacking in the Paraguayan Chaco.

## Material and methods

### Study Area

**Fig. 1:**
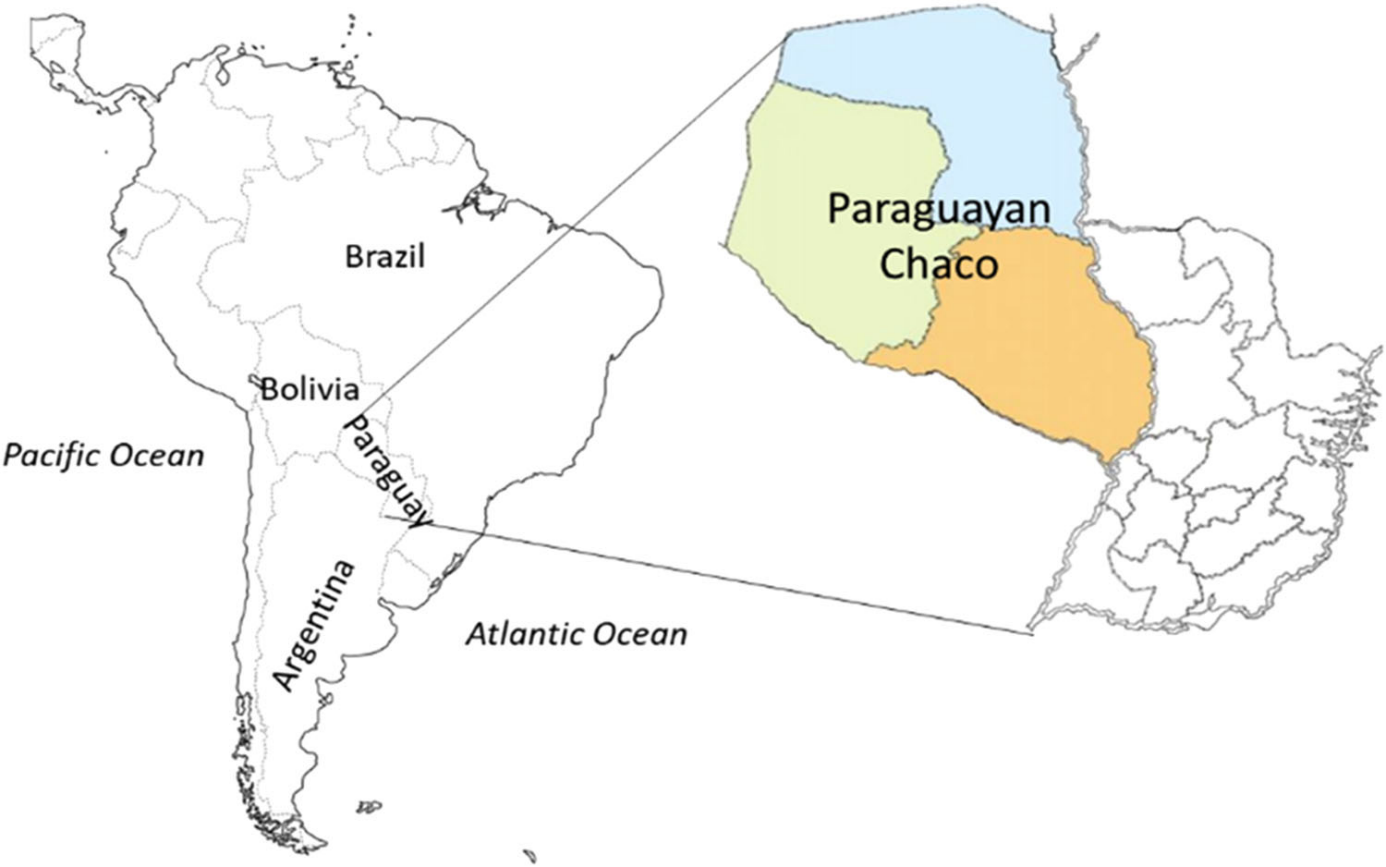
Map of Paraguay and the Paraguayan Chaco region

The western region of Paraguay, or the Paraguayan Chaco, comprises 61% (246,925 km^2^) of the Paraguayan territory (Fig. 1). This region is characterised by a flat topography, which presents a gradual increase in its relief, ranging from 80 m.a.s.l. in the eastern section to 400 m.a.s.l. in the west. The average temperature is around 25 °C. The warmest areas are concentrated in the northeast of the region, with maximums reaching 40 °C. The rainy season is the warmest and lasts from October to April. The highest levels of rainfall are found on the eastern side of the Chaco (1400 mm), adjacent to the Paraguay River. These values gradually decrease, reaching the minimum in the north-western region (less than 500 mm) (REDIEX, 2009). Consequently, the prevailing natural vegetation depends on the area. The eastern side of the Chaco is dominated by sub-humid and semi-deciduous forests. In the central and western zone, the vegetation is characterized by a mosaic of vegetation types composed of riparian, floodable and xeromorphic forests, different types of savannas and natural grasslands. Currently, due to the increase in agricultural and livestock activity in the area, natural and implanted grasslands and other crops (i.e., peanuts, sesame, spurge, sorghum, chia, safflower, wheat and oats) have gradually replaced natural vegetation, causing further fragmentation of the Chaco forests (Mereles and Rodas, 2014; Baumann et al., 2017; Gill et al., 2020; Da Ponte et al., 2022).

In the Paraguayan Chaco, 25% of the lands are under a conservation regime, as part of the National System of Protected Areas (SINASIP). The largest protected areas are located mainly in the north and most of them are in the public domain (93%), with the remaining 7% being private (Gill et al., 2020). In part of this protected area, land use changes are allowed for the development of agricultural or livestock activities, ensuring the conservation of 50% of the area in natural conditions or with minimal anthropic alterations. In the rest of the Paraguayan Chaco (75% of the total area) there are several laws issued to protect natural and forest resources, particularly law 542/95, which requires that 25% of the surface area of farming estates remains forested. Moreover, the conditions that must be met for planned deforestation to be approved are becoming more stringent, so that the right to clear cutting for cattle ranching requires that this change in land use is done in accordance with a silvopastoral system (Veit and Sarsfield, 2017).

Currently only 3% of Paraguay’s population resides there (INE, 2022). The population density is exceedingly low (0.86 people/km^2^; INE, 2022), with an increase in recent decades (54% between 2000 and 2020). The land of the Paraguayan Chaco sheltered uncontacted indigenous peoples until mid-19th century, when their lands were almost entirely sold by the government to Brazilian, Argentine, English, and French companies following the War of the Triple Alliance (Vazquez, 2007; Vazquez, 2013). It was at this point that the ‘quebracho’ industry started developing, declining after 1950 (Vazquez, 2007). Since the mid-19^th^ century, due to the need to populate the Chaco, several attempts were made to find foreign settlers. After several unsuccessful attempts, in the 1920s, the Paraguayan government granted certain privileges for the establishment of Mennonite colonies arriving from Canada, Russia, and Germany, who settled and lived alongside the indigenous communities. The enterprising nature of the Mennonites and their agrarian culture helped the development of agriculture in this area (Vazquez 2013). Thus, in the 1960s, the introduction of more productive pastures and the improvement of infrastructures with the construction of the Transchaco route, facilitated the expansion of dairy farming in the region, which began to supply milk to almost all Paraguay, and even some of the neighbouring countries (Vazquez, 2013). In the 90 s, the development of cattle farming for meat production began, with investments being made not only by the Mennonites, but also by Paraguayans from the east and foreign entrepreneurs from neighbouring countries, particularly Brazilians, Uruguayans, and Argentines, who purchased large expanses of land (le Polain de Waroux, 2019). The arrival of these entrepreneurs is related to the economic situation, and restrictions on deforestation legislation in these neighbouring countries and in the eastern region of Paraguay (Baumann et al., 2017; le Polain de Waroux, 2019).

At present, the inhabitants of the Paraguayan Chaco are mainly indigenous peoples of various ethnic groups (e.g., Ayoreo, Guaicurú) representing approximately 30% of the population (Gill et al., 2020), the descendants of Mennonite immigrants, and in less quantity the local mestizos “Paraguayans” (Mereles and Rodas, 2014). The region’s economy is mainly based on forestry and livestock, specifically beef- cattle ranching. The Paraguayan Chaco accounts for 45% of Paraguay’s cattle population (6.3 million head and 8,003 ranches; SENACSA, 2023). In the region, the rate of increase in the bovine population has accelerated in recent years, such that between 2010 and 2020, the volume of cattle rose 24.0% (SENACSA, 2023). Cattle farming is mainly for meat production, is carried out on large ranches, feeding is based on pasture and is highly export oriented (Milán and González, 2023). Dairy production has been losing relative importance (Gill et al., 2020) and is mainly destined to supply the Paraguayan market.

### Data Collection and Statistical Analysis

Q methodology was used to characterise the different discourses that coexist in the Paraguayan Chaco concerning the development of cattle ranching and derived deforestation. This methodology allows examining the inherent subjectivity existing in all social conflicts in a structured manner (Addams, 2000; Nijnik et al., 2014; Zabala et al., 2018). Q methodology is exploratory and semiquantitative, combining benefits of quantitative and qualitative approaches and, although Q methodology is based on factor analysis, it focuses on similarities between individuals and not on similarities between variables (Webler et al., 2009; Zabala et al., 2018), therefore factors depict the opinion of an archetypical interviewed who would best represent that factor, although they do not necessarily describe any specific real interviewee (Zabala et al., 2017). It has been widely used in various disciplines and scopes (Cairns et al., 2013; O’Riordan et al., 2016; Pereira et al., 2016) to scrupulously explore social phenomena being characterized by the existence of a multiplicity of interests, opinions and values in conflict. The Q methodology helps to identify and characterize the different patterns of thought or discourses that coexist on a given issue under debate, and this identification needs not be based on a preliminary hypothesis (Zabala et al., 2017). Q has considerable potential to help identify areas of consensus and disagreement around key topics, which can then be used to assess management alternatives, resolve conflicts, appraise policies, or facilitate critical reflection (Zabala et al., 2018).

**Fig. 2:**
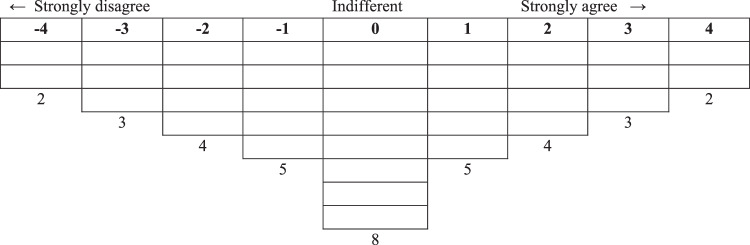
Grid employed for Q sorting the 36 statements by the key informants

The different steps that were followed in the application of the Q methodology are as follows. First, identifying study participants. In our case, local agents directly or indirectly related to the expansion of cattle ranching in the Paraguayan Chaco. The criteria employed to select the key participants was the maximization of the diversity of experiences and views concerning the issue at stake. This included: technical advisors with different profiles (veterinarians, environmental consulting and supplier industry workers), managers of cattle farming companies, ranchers (small and medium-scale ranchers <500 Livestock Units: 3 and large-scale ranchers >500 Livestock units: 3; one of them being the president of a producer association), government officials (veterinarians and environmental consulting), environmental activists, and researchers of the fields of animal production and environmental sciences (Table 1). In all cases, these were people who had a strong opinion on the subject of the study. Some of these people were contacted directly because of their social relevance and through them, the rest were contacted. Second, a first round of interviews was conducted to a sample of key informants. The interviews were conducted face to face and in Spanish by the second author of the work, both in Asunción and in the territory of the Paraguayan Chaco. Both in these interviews and in those that followed (step four), the participants were informed of the objective of the study and that their anonymity would be guaranteed. They lasted approximately 1.5 h and were held in 2018. Following the recommendation of Barry and Proops (1999) the sampling process was stopped when in the process of interviewing new views and experiences stop emerging. Finally, a total of 27 participants were considered. At this step, the questions that were addressed in the interviews were: how do you perceive the expansion of cattle ranching in the last years? What do you believe are the advantageous and detrimental effects of this expansion? Is the current legislation framework adequate to monitor the activity and to minimize potential unwanted effects? Is this expansion sustainable? What risks and threats do you see in it? Third, based on the responses obtained in the previous step, an initial sample of 185 statements were extracted, which were thought to represent the wide diversity of notions and ideas being suggested by the key participants on the issue at stake. From these initial sample, a reduction of statements was conducted by the researchers. In the process, an attempt was made to ensure that the remaining sentences represented all the ideas that emerged in the first phase of the interviews. To minimize the loss of diversity of ideas, all initial statements were grouped by domains (socioeconomic, environmental, and governance) and topics (e.g. beef cattle management, climate change, aquifers, soil compaction, implanted pastures, short and long-term effects, environmental legislation), so that the themes were not mutually exclusive. Subsequently, from all the statements that had the same tags, the most relevant ones were selected, so that, after reduction, statements from all domains and topics were maintained. The final selection was 36 statements. Fourth, a second round of interviews was conducted with the same (27) key informants who participated in the first round. In this case, the interviewees were asked to read the 36 statements obtained from the previous phase and place each one in a box on the grid (Fig. 2). That is, the interviewee must place each statement in a box, which means classifying each statement in relation to the other statements. The result of each interview conformed a Q sort. This classification presents a quasi-normal distribution, and it is assumed is a good representation of the position of the participant. Fifth, the 27 Q sorts obtained in the previous step were analysed by means of inverted factorial analysis (Principal Component Analysis), yielding various factors (Zabala et al., 2017). Varimax rotation was then applied to these factors, which resulted in each participant being associated with only one factor to simplify interpretation. Following the recommendations of Cairns et al. (2013), a solution was sought that maximised the explained variance and the number of participants that loaded significantly with a single factor, and minimised the number of participants that did not load with any factors. The free software package PQMethod 2.35 (Schmolck, 2014) was used to carry out the statistical analysis. Finally, these factors were interpreted as ideal Q sorts that we assume epitomize the essence of the different coexisting discourses.

**Table 1:** Participant profiles and their loadings on each discourse

Participants	Age	Gender	Environmentalist	Business	Resigned	Possibilists
Cattle rancher (SM-s)^a^	34	M	0.7341*	0.0371	0.1015	0.2525
Cattle rancher (L-s^a^, President farmers’ association)	50	M	−0.0974	0.7100*	−0.0529	0.0953
Farm manager	35	M	0.1108	0.7727*	−0.2395	0.0240
Research professor	48	M	0.5194*	0.3250	0.2309	0.3198
Technical advisor (supplier industry)	43	M	0.0733	−0.0411	0.5045*	0.1823
Technical advisor	35	M	0.3582	0.1424	0.4042	0.3449
Technical advisor (veterinarian cooperative)	55	M	0.1923	0.6902*	0.1276	0.1098
Technical advisor (environmental consulting)	35	M	0.4647*	−0.0728	−0.0892	0.3269
Technical advisor (veterinarian cooperative)	49	M	−0.0904	0.8145*	0.1210	0.0069
Technical advisor (environmental consulting)	35	M	0.5372*	−0.0793	0.0132	**0.4758**
Government official (veterinarian)	45	M	−0.1377	0.8343*	−0.1534	0.2534
Government official (environmental consulting)	41	F	−0.2721	0.3946	0.0719	0.6319*
Technical advisor (supplier industry)	33	M	0.1952	0.0397	0.0258	0.7263*
Cattle rancher (L-s)	43	M	0.3413	0.3001	0.0792	0.5587*
Farm manager	38	M	0.1265	0.2494	0.7850*	−0.1372
Cattle rancher (L-s)	65	M	0.8154*	0.0898	0.1187	−0.0892
Research professor	33	M	−0.0119	−0.0741	0.6727*	0.1160
Cattle rancher (SM-s)	42	M	−0.0939	−0.0115	**0.4491**	0.5061*
Government official (veterinarian)	42	M	0.1976	**0.4606**	0.1275	0.5617*
Researcher (veterinarian)	35	M	0.5845*	−0.2036	0.1815	0.1600
Technical advisor (supplier industry)	31	F	0.3706	−0.0099	0.0553	0.6667*
Researcher (veterinarian)	31	F	0.5645*	−0.0183	0.1919	0.2305
Farm manager	60	M	0.2418	−0.0772	0.5611*	−0.0849
Cattle rancher (SM-s)	61	F	0.4943*	−0.1567	−0.0914	**0.4493**
Research professor	33	M	−0.3235	0.6194*	0.3170	−0.2045
Professional environmental organization	36	M	**0.5546**	−0.5745*	0.0286	0.0403
Professional environmental organization	30	M	0.8323*	−0.0846	0.0485	−0.0860
**Percentage of explained variance**			**17**	**16**	**9**	**12**

^a^SM-s: Small and medium-scale ranchers <500 Livestock units; L-s: Large-scale ranchers >500 Livestock units;

*Indicate the defining Q sorts, which are those carrying significant weight in each discourse; The Q sorts that load significantly (*P* < 0.01) but are not defining ones are in bold.

## Results

Four different discourses were identified as making sense the wide diversity of interests, opinions and values coexisting and clashing in the Paraguayan Chaco as regards the present expansion of cattle ranching and the derived deforestation, namely: the Environmentalist discourse, the Business discourse, the Resigned discourse, and the Possibilist discourse. All participants except one loaded significantly on at least one discourse (Table 1). This, jointly with the 54% of the explained variance of the model, indicates the good explanatory quality of the existing diversity of point offered by the four-discourses model we propose. The interpretation of discourses is based on the statement scores shown in Table 2. The statement scores value represent the weighted average of the values that the participants most closely related to the factor give to a statement, suggesting how the archetypical participant for each factor would sort the statements (Zabala et al., 2017).

**Table 2:** Statements scores for each of the four extracted discourses

Statements	Environmentalist	Business	Resigned	Possibilists
1. One of the main benefits of cattle ranching is the great demand for manual labour, both qualified and unqualified.	−2	0*	3*	−1
2. The expansion of cattle ranching is highly beneficial to Paraguay in economic terms, but in terms of sustainability important adjustments need to be made, together with coherent public policies.	**2**	**1**	**−1**	4*
3. Cattle ranching has improved the state of biodiversity in the Chaco. The cattle ranches shelter more biodiversity than the closed native forests.	−3	4*	−3	0*
4. In recent years, the advance of the livestock frontier has greatly impacted the quality and quantity of water in the region.	0	0	−3	−2
5. I consider it greatly beneficial. Thanks to the development of cattle ranching the access routes, mobile phone coverage, and electric network have been improved in communities that previously lacked such services.	1	3*	−1*	2
*6. A great improvement in the quality of the animal-source food produced is under way, which is perceivable by the presence of the Paraguayan product in international markets that are constantly more demanding*.	4	2	4	2
7. Cattle ranching has great margin for growth, which would increase even further the opportunities for employment creation, investment attraction, foreign currency incomes and taxes.	2	3	4	3
*8. One of the main benefits of the expansion of cattle ranching is the development of the agricultural industry: e.g. slaughterhouses and relevant suppliers*.	−1	1	0	1
9. Cattle ranching has caused uncontrolled deforestation and significant land degradation, and soil compaction.	1*	−4	−1*	−3
10. I do not see detrimental aspects in the expansion of cattle ranching, except for the activism and disinformation diffused in ever-increasing desperation by environmental NGOs.	**−3**	1	0	−1
11. The current environmental and forestry legislations are highly protectionist. If they were observed sustainability would be guaranteed.	−2*	4*	**1**	**−1**
12. More forceful actions are needed in the management of soils, water, and protective fringes with native forests and wider natural reservation areas.	1	−2*	1	2
13. The practices being conducted by cattle ranchers since the very beginning of this activity in the region have comprised numerous elements of sustainability, particularly in terms of gene pool preservation and sustainable practices of water storage and capture, and conservation of pastures.	−3	3	−2	3
14. The soil has particularly good characteristics to produce pastures. It is rich in phosphorus and shows good capacity to retain rainwater.	0	**2**	−1	0
15. Cattle ranching in the Chaco is highly productive, but it runs a high risk of becoming unsustainable over time.	−1	−3	−2	−4
16. The current environmental regulations do not take the right approach towards sustainable production and should thus be revised. This is also the case of the public policies on sustainable farming.	0*	−2	−2	3*
17. Cattle ranching has a large dependence on natural resources. The need to satisfy the demand for food often leads to overexploitation of natural resources and environmental deterioration.	3	−1	3	0
18. The overexploitation of natural resources, especially soil, is already showing signs of degradation, such as barren patches, impoverished soils, and lower quality pastures.	**2**	**0**	**−2**	**1**
19. Most cattle ranchers do not consider medium- and long-term needs when it comes to secure a sustainable agroecosystem over time.	4	**−2**	2	**−3**
20. The Chaco could be sustainable, but in some cases regulations and recommendations of best practices are not followed. This is particularly the case of tasks for soil fertility restauration and erosion control.	2	0	0	1
21. The greatest threat is that in remote areas, because of the little capacity of the Government of surveillance, environmental and forestry regulations are not observed.	1	0*	2	**4**
*22. The main risk that could affect sustainability is the lack of dialogue and agreements between ranchers, environmentalists, and public institutions*.	0	0	−1	0
23. Local studies on the real impact of cattle ranching are urgently needed.	3*	1	0	0
24. The main threat of livestock expansion is that in the future there will be indiscriminate deforestation, in conjunction with the absence of rational grazing.	0	−1*	3*	1
25. The low cost of productive land is problematic. It is cheaper to purchase more land in need to be broken than to improve agricultural land.	−4	−4	2*	−1*
26. One of the main aspects to be improved is the lack of transparency in the value chain of veal.	**3**	2	1	−2*
27. Sustainability in the Chaco is in danger because a large part of the farming/infrastructural projects being built do not respect the hydrological dynamics, or the connectivity of natural ecosystems.	1*	−3	−3	**−2**
28. Environmental legislation is inadequate as it was designed for the Eastern region, whose dynamics and problems are quite different than those in the Chaco.	−1	−1	−4*	0
29. Climate change does not pose any risk to the Chaco, in the worst-case scenario temperatures may rise, which would not pose a problem in this region.	−4	0*	−4	−4
30. A possible risk derived from climate change is the lack of resilience of the cattle systems.	−1	0	0	−2*
31. The cultivation of Gatton Panic requires great extensions of treeless land, which could generate erosion and diminish biodiversity.	0	−2*	0	2*
32. Soy is a crop that is spreading in the Chaco Central and Alto Chaco, competing with cattle ranching for access to the most productive land.	−1	−1	1*	**−3**
33. Soybean cultivation is an activity with rapid financial returns, therefore this cultivation, in the future, could displace cattle ranching, as has already occurred in the eastern region.	**−2**	**−1**	**2**	**1**
34. In the Chaco, diversifying cattle farming fields with soybean cultivation would not only be financially advantageous, but it would also break the monoculture, forcing the producers to maintain soil fertility.	−2*	2*	1*	−1*
35. Implanted pastures, such as Gatton Panic, have high productivity but bring about a high extraction of nutrients and high levels of soil compaction due to high stocking rate.	0	−3*	0	0
*36. Political pressures are exerted to change laws in benefit of certain groups and to favour the expansion of the livestock frontier*.	0	1	0	0

In bold distinguishing statements for each discourse (*P* < 0.05). * indicates significance at *P* < 0.01. Consensus statements on italic.

### The Environmentalist Discourse

This discourse accounts for 17% of the variance, and a total of 37% of the key participants interviewed adhered to it significantly. Profiles associated with this discourse are two technicians that carry out environmental advisory, two research professors, two workers of an international environmental organisation, and three producers (2 small and medium-scale and 1 large-scale rancher), who show notable environmental conscience (Table 1). Proponents of this discourse are critical and concerned about the current situation as well as with the future uncertainties the expansion of cattle ranching, and its effects might trigger. This view claims that an overexploitation of natural resources is taking place in the region, fundamentally led by the expansion of cattle ranching. They point that there is a strong need of cattle ranching to incorporate the consideration of the long-term sustainability of the activity.

The advocates of the Environmentalist discourse claim that the impoverishment of the soils, as well as loss of biodiversity, are already observable in many areas (Table 2, #3, 17, 18 and 19). This discourse is convinced that the present cattle ranching practices are unsustainable in terms of multiple dimensions: crosses with foreign breeds, water and pastures conservation, and biodiversity preservation (Table 2, #13). This discourse is the only one strongly indicating the unsustainability of the expansion of cattle ranching in terms of deforestation, and ecosystem and land degradation (Table 2, #9, 27). Proponents of this discourse consider that current legislation is not suitable to ensure sustainability, so changes in current policies should be made, as well as improving transparency in the value chain of veal (Table 2, #2, 11, 26) and conducting further studies to better comprehend the impact of the expansion of cattle ranching (Table 2, #23). Concerning the socioeconomic dimension, this discourse identifies some advantageous aspects in the cattle ranching development, although, in opposition with the Resigned discourse, do not attribute to this activity a relevant capacity of employment creation (Table 2, #1).

### The Business Discourse

The Business discourse accounts for 16% of the variance. A total of 22% of the key informants interviewed adhered to it positively and significantly, a case presents a negative correlation indicating rejection of that agent for this discourse. The profiles that are linked positively to this discourse are the two technicians that work for a producers’ association, one producer (a large-scale rancher that is the president of a beef cattle farming association), a research professor who also works as a veterinarian advisor, a farm manager and an official government veterinarian. In other words, the majority of participants who have a technical-production profile (Table 1). The followers of the Business discourse are resolute defenders of the cattle ranching expansion. The advocates of the Business discourse only perceive advantages and opportunities in the expansion of cattle ranching in the Paraguayan Chaco. The key idea kept by the advocates of the Business discourse is highlighting the multiple benefits that cattle ranching, associated with the cultivation of implanted pastures such as Gatton panic, is bringing about into the Paraguayan Chaco and into the country (Table 2, #3, 5, 31, 35). This discourse claims that the Paraguayan Chaco was previously unused and thus was not making any contribution. The development of cattle ranching is the opportunity of incorporating the Paraguayan Chaco into the economy, as it is now a source of new economic opportunities and wealth-generation activities.

The advocates of the Business discourse see a wide margin for further growth (Table 2, #7) and they also claim that the existing implementations of the environmental and forestry legislations are protectionist enough and adequate to guarantee the sustainability of the activity and the region as a whole (Table 2, #11, 12, 21, 27). In this sense, they do not see any risk of deforestation linked to the development of cattle ranching or soybean cultivation (Table 2, #9, 24, 34). In fact, both activities are seen as largely beneficial for the region. In line with this, the sustainability of the system in the medium- and in the long-term is not of much concern by the advocates of the Business discourse (Table 2, #15, 19).

### The Resigned Discourse

The Resigned discourse accounts for 9% of the variance. A total of 15% of the interviewees adhered to it significantly. The profiles associated with this discourse are mostly managerial. This is a technical advisor working for an agricultural input company, two ranch managers and a research professor (Table 1). The advocates of this discourse are characterized by accepting the present situation of the expansion of cattle ranching that triggers some unwanted effects. They see the associated environmental impacts as a price to be paid for the development of the region. Conformism is largely characterizing the general attitude show by this discourse. They are convinced that the prevailing logic of development follows inextricably an economic reasoning and that there is nothing to be done to incorporate within it other social and environmental concerns.

The Resigned discourse is characterised by the opinion that cattle ranching in the Chaco brings many benefits, as also claimed by the Business discourse. Although it is aware of the existence of some detrimental effects, it believes that these are inevitable and are the price to be paid for developing (Table 2, #17). The followers of this discourse do not believe that the expansion of the livestock frontier entails any overuse of natural triggering soil and pasture degradation (Table 2, #9, 18) they also highlight the employment creation it entails (Table 2, #1). However, the proponents of this discourse also identify the existence of unwanted consequences, as it is the case of biodiversity loss or lack of infrastructure development (Table 2, #3, 5).

Concerning public policies, the Resigned discourse sees them as largely adequately implemented in the region (Table 2, #2, 11, 16, 28). Thus, it is not believed that significant adjustments or changes in policies were needed to further guarantee sustainability. The risks the advocates of this discourse see are derived fundamentally from the possibility that future indiscriminate deforestation and overexploitation might take place in the future if the expansion of cattle ranching further intensifies (Table 2, #24). In this regard, it is considered that the low price of land is favouring the expansion of cattle ranching and this might pose a risk in the close future, particularly in terms of soil degradation (Table 2, #25).

### The Possibilist Discourse

This discourse accounts for 12% of the variance. A total of 22% of the key informants interviewed adhered to it significantly. Profiles associated with this discourse are two producers (1 small and medium-scale and 1 large-scale rancher), two technicians that work in supply industries, and two government officials (Table 1). The followers of the Possibilist discourse are strongly advocates of the status quo with some few amendments, particularly in the domain of environmental surveillance. This discourse claims that a fundamental role should be played by public policies in conducting adequate scrutiny of the cattle ranching activities and its consequences. The advocates of this discourse see cattle ranching as an activity largely compatible with the conservation of natural resources as well as greatly beneficial to the Paraguayan economy. However, they also underline the need to carry out some significant adjustments in the regulations being implemented to guarantee the sustainability of cattle ranching and of the region in the long term (Table 2, #2). The advocates of the Possibilist discourse consider that the practices being conducted by cattle ranchers have comprised since the very beginning of this activity in the region numerous elements of sustainability. Consequently, they believe that at present cattle ranching is not running high risk of becoming unsustainable (Table 2, #13, 15, 19), keeping that the is still margin for further expansion of cattle ranching in the Paraguayan Chaco (Table 2, #7). They do believe, however, that current environmental regulations should be amended and more effort should be devoted to surveillance to guarantee their observation, particularly in remote areas (Table 2, #11, 12, 16, 21).

### Domains of Consensus Among the Discourses

Exists consensus among the four discourses in several domains (statements with *P* < 0.01; Table 2). All discourses agree that remarkable improvements have been accomplished in the quality of the bovine production in the region in the last decades. Thus, Paraguay has become one of the leading global exporters of veal (Table 2, #6). There is also consensus in statements #8, #22, and #36, but in these cases, the statements are scored −1, 0, or 1, which would indicate that all the discourses are indifferent to the development of the agricultural industry (slaughterhouses and related industry). They are also indifferent to the claim that the lack of dialogue and agreements between producers, environmentalists, and public institutions put the activity at risk, or that there are pressures put on politicians to change laws to benefit a specific group.

The results also point the existence of some domains in which although there is no consensus, it is apparent observed the lack of conflicting views. This is the case of the consideration that cattle production still has margin to grow, being the “Resigned” who highlight this aspect (Table 2, #7). The results also underline that, some very important issues for discourse 4, such as the need to conduct notable adjustments to enhance the sustainability of the system, and that this goes largely in consonance with the development of coherent development policies, could be promote increasing overall satisfaction without decreasing the satisfaction of any one discourse (Table 2, #2, 21).

## Discussion

The coexistence of the four discourses identified illustrates the complexity and the multiple values, beliefs and interests that cohabitate and occasionally clash in the domain of the expansion of ranching in the tropics. The consideration of this complexity is particularly important to understand the phenomenon of cattle expansion in this zone and in determining both the type of land management conducted and the implementation of effective public policies (Steelman and Maguire, 1999) that regulate the expansion of cattle ranching and reduce deforestation rates in the Paraguayan Chaco (Pinillos et al., 2021).

Similarly to what Huaranca et al. (2019) and Zepharovich et al. (2020a, b) observed in Argentinean Chaco, our results point that the explanatory dichotomy between conservation versus production is an oversimplification. Also, the absence of a relationship between the different categories of interviewed agents and the different discourses reveals the false perception that each group of agents will exclusively defend their interests, identifying the profile of an agent with certain ideas, as it has also been reported by Huaranca et al. (2019) and corroborating the hypothesis of Brannstrom (2011), that environmental discourses are not attributable to the structural position of an actor within a socio-economic system, but rather are deeply held and contested truths that represent fundamental ideas about environmental or social processes. For example, technical advisors loaded on the four discourses, and cattle ranchers and research professors loaded on three discourses. Only the professionals of an environmental organization and the researchers (veterinarians) loaded exclusively on Environmentalist discourse.

The advocates of the Business discourse are convinced that the expansion of cattle ranching can only bring prosperity into the region. This discourse is based on the values called “agriculturalist” by Hoelle (2018), which represent an anthropocentric and instrumental view of nature in which the ability to provide food, property and profit are valued over environmental concerns. The Business discourse is very close to the “agricultural production for a globalized economy” perspective, reported by Huaranca et al. (2019), in terms of both, its scepticism about the severity of the environmental unsustainability, and its position stressing that cattle production strongly “contributes to development and poverty eradication, and thus benefits society at large” as pointed by Zepharovich et al. (2020a), which identified forest with poverty. Moreover, proponents of this discourse align themselves with the group of “large farmers” reported by Cáceres et al. (2020), with the “Development” discourse identified by Zepharovich et al. (2020b) and with the “Agribusiness perspective” according to Córdoba and Zepharovich (2022), by valuing soil fertility and water retention (#14) as important attributes of the ecosystem that they want to appropriate. This discourse is strongly anti-conservationist and solely focused on production. Unlike the exponents of the Business discourse, the advocates of the Environmentalist discourse claim that the expansion of cattle ranching is intimately linked to a set of environmental and social problems that cannot be disregarded, such as indiscriminate deforestation, overexploitation of water resources, soil compaction and erosion, loss of biodiversity, emission of greenhouse gases, and lack of transparency throughout the value chain, which only benefits a few actors involved in beef-cattle ranching. The Environmental discourse is close to the “critical environmentalism” perspective, identified by Huaranca et al. (2019), since according to its view cattle production and the derived deforestation generate dramatic environmental impact. The Environmental, Resigned and Possibilist discourses reflect, to different degrees, values termed “Pro-forest” by Hoelle (2018). These values are aligned with ideas and policies ranging from total forest conservation to more respectful and sustainable agricultural and or forestry uses.

The Resigned and Possibilist discourses, while not rejecting the expansion of cattle ranching in the Chaco, recognize some of its detrimental consequences. Both discourses differ fundamentally in their different attitude towards the phenomenon of cattle farming expansion. While the proponents of the Resigned discourse show a conformist and passive attitude, the supporters of the Possibilist discourse present an active attitude. Supporters of the Possibilist discourse believe that the detrimental effects could be avoided by increasing surveillance and issuing some policy measures. The Possibilist discourse is largely coincidental with the “environmental justice and inclusive dialogue” notions of Huaranca et al. (2019), mainly in considering that the government should play a central role in enhancing the coexistence of cattle production and nature conservation. It also presents certain similarities with the group called “land use planning enthusiasts” in the study conducted by Pinillos et al. (2021) in the Brazilian Amazon, in agreeing that new policies and approaches to land-use planning are needed.

In view of all this, we believe that the four coexisting discourses identified can be largely explained by the dissimilar positions held by the proponents of the different discourses on three key domains: first, the amount of socio-economic benefits the expansion of cattle ranching is perceived brings about in the region; second, the amount of environmental impacts the expansion of cattle ranching and the derived deforestation brings on in the region; and, third, the degree to which an active intervention from the side of policy making to regulate the expansion of cattle ranching and to minimize possible detrimental effects is perceived as necessary in the region.

In relation to the amount of socio-economic benefits that the expansion of cattle ranching in the region is perceived to brings, all the discourses agree on the improvements in the quality of the veal produced, which positions Paraguay in the international market, as evidenced by the fact that Paraguay is the ninth largest exporter of bovine meat in the world (USDA, 2023). There is also wide agreement in pointing that cattle ranching in the Paraguayan Chaco has margin for growth. The scope for agreement is smaller concerning the relationship between the expansion of cattle ranching and further development of infrastructures, or concerning the capacity of the expansion of cattle ranching of employment creation, with only the Resigned discourse pointing this. These divergences reflect the fact that Paraguay, despite undergoing notable GDP growth in the last decades, with a relevant contribution in it by the expansion of cattle ranching, has one of the greatest inequality rates all over Latin America, with particular implications in the distribution of land property (Veit and Sarsfield, 2017). Ávila and Portillo (2017) reported larger inequality and poverty rates in those departments of Paraguay where cattle ranching and soy production were more expanded. In fact, two of the three departments that comprise the Paraguayan Chaco - Presidente Hayes and Boquerón - are among the five departments of Paraguay with the largest inequality indices. The third department that comprise the Paraguayan Chaco - Alto Paraguay - is one of the five departments with the highest levels of poverty and extreme poverty. These data indicate that the appropriation by large landowners of the natural wealth of the area (i.e. land, fertility of the soil, water) does not have a compensation in the inhabitants of the area and confirm that the notable development of large-scale industrial farming, with huge land-consuming farming estates, many of which are in the hands of multinational agribusiness enterprises and foreign investors, scarcely generates wider social benefits any advantageous effects for the indigenous peoples and part of the local inhabitants, similar to what Margulis (2004) observes in Amazonia and Cáceres et al. (2010; 2020) in Northern and Western Córdoba (Argentinian Chaco). In fact, a good deal of the indigenous peoples has been dispossessed of their lands and displaced from their settlements (WWF, 2016; Vindal and Rivera-Andía, 2019). In addition, when they are employed by the farming companies to clear land or to raise cattle, they tend to be subject to precarious conditions, largely akin to serfdom (Ortega 2013; Veit and Sarsfield, 2017). All this seem to point that the expansion of cattle ranching in the Paraguayan Chaco goes largely associated with the continuation of the extractivist economic model that was imposed by the General Bernardino Caballero on the Paraguayan Chaco in the middle of the 19th Century, after the War of the Triple Alliance (Ortega, 2013).

Regarding the amount of environmental impacts the expansion of cattle ranching and the derived deforestation brings on in the region, the four discourses show notable disagreement, in term of both the perceived impacts of the expansion of cattle ranching and the interrelated causes. The Business discourse is the only one highlighting that the expansion of cattle ranching is not triggering uncontrolled deforestation causing environmental impacts, in terms of land degradation and soil compaction, or biodiversity loss. The improvement in biodiversity due to the expansion of cattle ranching (Table 2, #3) is a rather contentious idea being held by the advocates of the Business discourse. The existing debate on the consequences of the expansion of cattle grazing and deforestation on natural ecosystems is largely entrenched (Schieltz and Rubenstein, 2016; Mazzini et al., 2018), and specifically on biodiversity (Perfecto and Vandermeer, 2008). The point held by the proponents of the Business discourse is unscientifically and could be related to observations by ranchers that wildlife tends to concentrate around water points on their ranches, and infer that there are more animals. This vision is intentional and could be based on several studies that report very heterogeneous responses depending on the forest ecosystem in question, the animal species being raised and the specific farming practices being conducted (Torres et al., 2014; Grau et al., 2015; Marinaro and Grau, 2015; Mazzini et al., 2018).

Regarding deforestation, proponents of the Resigned discourse, with his economicist vision, see the low land price as a trigger of land degradation. The existing connection between cheap land and the expansion of cattle ranching and land degradation has been reported in different areas of South America (Zarrilli, 2010; Margulis, 2004; Soto and Gómez, 2012; le Polain de Waroux et al., 2016). The proponents of the Resigned discourse, they also point the existing interaction between the expansion of soybean production and the expansion of cattle ranching, the cause being, also in this case, the price relationship. The risk is that it will occur as in the Eastern region of Paraguay and in other nearby areas in Latin America, where initially were the expansion of cattle ranching and their pastures the main cause of deforestation, while later was the rise in the price of soy in relation to veal what generated incentives to convert these pastures into land for soybean cultivation (Nepstad et al., 2006; Barona et al., 2010; Fehlenberg et al., 2017; Parente et al., 2019; Henderson et al., 2021).

Aspects such as the need in the region for a more active intervention from the policy-making side to regulate beef-cattle expansion and minimize possible detrimental effects, as well as what type of legislation is most adequate, and what is the best way to enforce this regulation, are questions being answered largely dissimilarly by the four discourses identified. The opinions range from the Business discourse that maintains that the present environmental and forestry regulations are too protectionist, to the Environmentalist discourse that claims the existing regulations are largely not enough to prevent land degradation and biodiversity conservation; and the Possibilist discourse that considers that the present environmental regulations are not well fitted to foster environmentally-aware cattle ranching, they also defend that the audit mechanisms the authorities implement lack effectivity, especially in the most remote areas, where the capacity for enforcement by the public organisms in charge (e.g. National Forestry Institute, INFONA) is quite limited, as is their technical capacity to monitor the territory in a systematic and continuous manner (Veit and Sarsfield, 2017; Salinas et al., 2023). However, an important finding of this work is that despite these differences, providing more forceful actions in the management of soils, water and wider natural reservation areas, would have the support of all the discourses except the Bussines, also improving law enforcement could be promote by the Paraguayan administration without any discourse against. Another aspect to highlight is that the position of the Environmentalist and Possibilist discourses on this issue is quite close, so that an alliance between both discourses could facilitate the implementation of certain environmental policies. Moreover, the passive attitude of the proponents of the Resigned discourse, although it would not align them with the Environmentalists and Possibilists to promote restrictive measures, would not make them active opponents either.

Several studies have provided significant evidence of reductions in deforestation in South America’s soy and cattle frontiers upon implementing certain public policies, such as establishing positive incentives for landowners who are making the transition to sustainable, low-deforestation production systems, together with the expansion of protected areas, monitoring systems, field inspections, and sanctions (Nepstad et al., 2014; Börner et al., 2015; Sousa, 2016; Nolte et al., 2017). These authors observed that the effectiveness of the protection measures depends on elements, such as the type of dissuasion method, the governance system and political will, the size and location of the ranch, and the state of the property rights to land. In the case of the Paraguayan Chaco, the combination of forests being mostly privately owned, the existence of political corruption, and the difficulty of access into many areas, comprise notable obstacles for the compliance of the existing anti-deforestation regulations (le Polain de Waroux et al., 2019; Salinas et al., 2023). Nevertheless, Nolte et al. (2017) reported that in Paraguay the degree of non-compliance of the existing anti-deforestation regulations was lower than in neighbouring countries. This could be because the expansion of cattle ranching in the Paraguayan Chaco is more recent, and there is still enough land available, so the price of land is lower than in other neighbouring areas, what has allowed the livestock farming estates to expand by purchasing new land. However, if land price increases in the future, the incentive to comply with the regulation may decrease. That is, as the cost of observing regulation increases, if surveillance and sanctions do not increase accordingly, the regulations might decrease in effectiveness (Börner et al., 2015). It is also possible, as Angelsen (2010) and le Polain de Waroux et al. (2019) point, that the effects of restrictive policy measures could induce intensification (technological improvements) in cattle production, as evidenced by the work carried out by Milán and González (2023) in this area. Therefore, these restrictive policies could have a rebound effect, the so-called “Jevon paradox”, as greater productivity is an incentive to increment the surface area of cattle farms enhancing thus deforestation. In this regard, Phalan et al. (2016), propose “active” land preservation instruments such as land-use zoning, land taxes and subsidies and voluntary standards and certification that reward good performance with market access and price premiums. These instruments could mitigate these rebound effects by linking yield increases to habitat protection or restoration. Two points of consensus observed in this study are the low relevance in the sector of the lobbying activity that could be exercised by some to bias policy measures issued in their favour and that it is feasible to reach agreements between cattle ranchers, environmentalist groups and public institutions, which could favour the establishment and enforcement of these instruments (le Polain de Waroux et al. 2018; 2019).

This Q study has allowed us to identify different perspectives on beef-cattle ranching in the Chaco by identifying areas of consensus and disagreement, which are key in policy formulation and conflict resolution (Durning, 2006; Zabala et al., 2017; 2018). However, it is important to mention some aspects related to the Q methodology that limit the results obtained. On the one hand, because it is not a purely quantitative or positivist methodology, as it requires active participation on the part of the researchers (e.g., purposeful choice of interviewees, selection of statements, interpretation of factors), the points of view obtained cannot be extrapolated to the whole population (Zabala et al., 2018). On the other hand, the inclusion of some additional stakeholder groups (e.g. slaughterhouse and ranch workers) could have revealed some additional perspective. Furthermore, the Q methodology does not allow us to know the relative distributions of the different perspectives obtained in this work in the community. To obtain this information, Webler et al. (2009) propose conducting a survey that asks a representative sample of the population the degree to which they agree with each of the discourses. As Davies and Hodge (2007) point out, this question is relevant from a political perspective since, a predominance in the community of Environmentalists, Possibilists and Resigned would facilitate the acceptance of certain environmental regulations. However, a predominance of Business could encourage the Resigned (who have a more passive attitude) to adhere to their discourse and therefore make it difficult for environmental legislation to be reinforced. Despite these deficiencies, the Q methodology has allowed revealing latent points of view and attitudes that otherwise are hard to identify.

The analysis of the results obtained raises different questions and future research. The environmentalist discourse highlights the need to carry out more studies on the real impact of cattle ranching in the region (for example, on biodiversity, water reserves, soil compaction), in addition, the preservation of forest areas also requires governance initiatives that revalue the forest. In this sense, research should focus on the implementation of agroforestry or silvopastoral systems that minimize the impact on the region. Another question that arises from the present work is what kind of policy orientation might be best accepted. Different policy instruments (e.g., prohibitions, incentives, regulations) will have different impacts and degree of acceptance, given the differences in motivations and interests among these groups (Davies and Hodge, 2007; Brannstrom, 2009). Results also point that to address the social dimension of this phenomenon, the opinions of other actors not directly related to cattle production, but affected by it (e.g. indigenous people, other small-scale owners affected by deforestation, people from social NGOs) should be incorporated. In this broader approach, it is possible to move from a local perspective, in which the opinions of the indigenous population and other inhabitants of the region are taken into account, to a broader vision that considers the population of Paraguay and neighbouring countries.

## Conclusions

This paper stresses that to better capture the complexity and context-dependent nature of the phenomenon of tropical deforestation, it is necessary to take into consideration the particular social. As shown in this paper through an exhaustive examination of one of the most active agriculture and livestock frontiers in the world, the Paraguayan Chaco, there is more variety than uniformity among stakeholders. Thus, four different discourses have been identified - namely, Environmentalist, Business, Resigned and Possibilist - that struggle to signify tropical deforestation in a way that better considers their worldviews and interests.

The exhaustive examination of the diversity within the social context in the particular case of tropical deforestation in the Paraguayan Chaco, also points the existence of three specific domains that should be carefully considered by policymakers to propose more effective measures: (i) the socio-economic benefits the expansion of cattle ranching brings about; (ii) the environmental impacts the expansion of cattle ranching and the derived deforestation brings on; and, finally (iii) the degree to which a need of policy intervention to regulate the expansion of cattle ranching and minimize tropical deforestation is seen as fundamental. In relation to this last point, issues such as enhanced surveillance of the effects of the expansion of cattle ranching, and the development of policy measures to minimize the unwanted effects being undergone mostly by those more vulnerable, could be promoted without major disagreements.

## Data Availability

No datasets were generated or analysed during the current study.
